# When Social Networks Meet D2D Communications: A Survey

**DOI:** 10.3390/s19020396

**Published:** 2019-01-18

**Authors:** Michele Nitti, George Alex Stelea, Vlad Popescu, Mauro Fadda

**Affiliations:** 1 Department of Electrical and Electronic Engineering, University of Cagliari CA, National Telecommunication Inter University Consortium, Research Unit of Cagliari, 09123 Cagliari, Italy; mauro.fadda@diee.unica.it; 2 Department of Electronics and Computers, Transilvania University of Brașov, 500036 Brașov, Romania; george.stelea@unitbv.ro (G.A.S.); vlad.popescu@unitbv.ro (V.P.)

**Keywords:** D2D communication, social, IoT, 5G

## Abstract

In the last few years, one of the main characteristics of the current technological development is the constantly increasing need for data exchange among various types of devices, both mobile and fixed. Within this context, the direct communications between devices has the potential to create new, location-based peer-to-peer applications and services, as well as to help offload traffic from the congested traditional cellular networks. The main hurdles for this kind of Device to Device (D2D) communications are throughput, spectral efficiency, latency and fairness. Most of these hurdles can be overcome by the use of the new Social IoT (SIoT) paradigm, of things and people involved together in the network, guided autonomously by social relationships following the rules set by their owners. This paper aims to investigate the state of the art of socially-driven D2D communications. Upon an initial analysis, we perform an in-deep literature investigation of the main directions in which social ties can improve D2D communication, draw conclusions and identify the research topics left open.

## 1. Introduction

The constantly increasing demand for local data services at increased data rates has led to the development of the D2D (Device to Device) communication paradigm that enables the direct communication of nearby mobile users without the advent of a higher level controller. The obvious advantages of this kind of communication are higher throughput and enhanced data rate, lower latency, fairness, improved spectral efficiency, and lower energy consumption [1]. These advantages are based on several technical challenges, including relay discovery and peer selection, communication mode selection, spectral resource allocation, interference coordination and management, channel allocation, and upgrade of existing infrastructures.

The novel D2D communication approaches have resulted in the last few years in innovative network architectures and applications that redefine the current state-of-the-art with respect to wireless connectivity, next generation cellular communications (e.g., 5G), Internet of Things (IoT), and also vehicle-to-vehicle (V2V) communications [2].

In the past few years, the idea of the convergence between D2D communications and social networks started to attract increased attention of the scientific community, which started to apply features from the social networking for increasing the performance of D2D communication. The most important social features that can be applied to the D2D communications are the social communities, social links and the concept of centrality. The quick success and vast diffusion of social networks, through platforms such as Twitter, Facebook and Instagram, generated vast amounts of data about the structure and dynamics of social networks encouraging researchers from various research areas to transversally apply “sociality” to their works [3].

However, the role of sociality is still not clear. Most of the currently published papers make use of social attributes coming from ties among persons. Nevertheless, the idea of things networked together with people has led to the Social IoT (SIoT) concept [4], where objects are able to establish social relationships autonomously, based on rules imposed by their owners. The resulting social network of objects is able to deliver a faster service and information discovery, by scanning a network of “friend” objects and is also able to manage in a more consistent way the received information.

Social networking for D2D communication refers to finding and exploiting the interaction patterns between the users, both people and objects, of the social network, to enhance the efficiency of the D2D networks based on proximity information.

The research on the combination between social-enabled networking and D2D communication is still at the beginning, therefore the most important research directions currently need to be identified and addressed. The aim of the present survey is to explore and understand the benefits of the integration of social-awareness with D2D communication to offer a clear picture of the current scientific efforts in this field. The survey identifies the key topics of D2D communication that can benefit from the social-enabled networking, and, for each of these topics, creates a taxonomy of the identified applications.

The paper is structured as follows: Section 2 gives a concise overview of the D2D concept through the lens of social networks and social ties and identifies the key issues to be investigated. In Section 3, the actual investigation is performed for three distinct issues, cataloging the current research efforts and highlighting the key social concepts that can be used to enhance the performance of D2D networks. Finally, in Section 4, the lessons learned from the performed surveys are put together to draw the final conclusions in Section 5.

## 2. System Description

As mentioned in the introduction, D2D communications were initially introduced to benefit from [2]:

- the proximity of devices for assuring higher bitrates, lower delays and reduced power consumption;
- the reuse of resources by simultaneously allowing both D2D direct-link communications and “infrastructured” communications (e.g., cellular or WiFi); and
- the use of a single link rather than involving resources for both an up- and a downlink.

The concepts of social networking can help in finding solutions and improvement for D2D communications in the communication domain where devices can help or be assisted before accessing a network or before starting a D2D communication and in the social domain where devices can create a social network governed by social relationships and events. Considering these two main domains, the following three key issues can be investigated:

- Relay discovery and peer selection: The efficiency of a D2D communication is mainly related to the neighbor discovery process that is necessary prior to the effective direct communication. Peer selection can be time and energy consuming if network support is not available.
- Communication mode selection: A device can select either an established transmission channel respecting the procedures and constraints of the used network infrastructure, or a D2D communication. Involving all the devices to take this decision can improve data transmission capacity and resource utilization.
- Spectrum resource allocation and management: D2D communications use the same spectrum resource and air interfaces as normal, structured communications. To use these resources more efficiently, D2D communications may be performed for example by reusing the same frequency channels, involving decision and scheduling methods for spectrum sharing.

**Figure 1:**
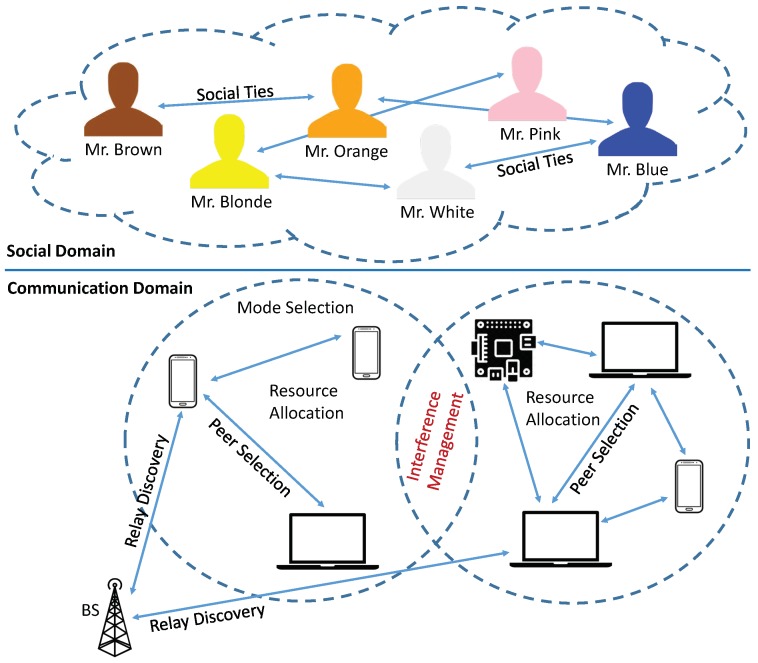
System design.

A schematic representation of a D2D communication environment, containing both the social and communication domains, is depicted in Figure 1. The previously identified key issues are inserted in the lower half of the schematic representation. It has to be said that, although identified, these three key issues cannot be clearly divided from the social point of view, considering the fact that some social concepts can interfere simultaneously with more than one issue.

The next section presents each of these three keys topics in terms of the solutions and algorithms proposed to improve D2D communications performance based on the social components.

## 3. How Social Ties Can Improve D2D Communications

### 3.1. Relay Discovery and Peer Selection

When a device wishes to establish a D2D communication, the network and/or the source user equipment (UE) has to find which are the possible peers that can be used as a relay by a peer selection procedure [5,6]. In the last five years, a social-aware approach has boosted the development of this procedure, such as the work proposed in [7], where the relay is selected according to the concept that two friends can serve as the feasible relay for each other, which is called the reciprocal cycles concept.

Concepts of social networking are also used in [8], where devices are aggregated in clusters based on human social ties; the cluster head, which has to act as the relay to communicate with the Base Station (BS), is then selected based on the centrality degree of the devices. A similar approach is proposed in [9], where social ties are used as incentives to enable D2D communication, where the stronger is the tie between two peers, the higher is the probability to select them as a relay.

In 2017, the authors of [10] introduced another perspective of social networks: they used attributes related to the service, such as the interaction information among the users and their contact history, to assess the reliability of the other users, as well as considered social attributes, i.e., users’ interests and their relative importance, to create dynamic clusters for the users with communication demand and then help them to select their neighbors. Recently, Du et al. [11] analyzed and suggested different mechanism on how to use the social features to optimize the network performance. In particular, they considered social features from the network perspective, such as the centrality of the devices, i.e., the devices’ frequency of forwarding data to other devices, and social features from the user perspective, where they relied on the credit, i.e., that the devices social relation is built based on trade for data forwarding, and reputation, i.e., the quality of forwarding or relaying a service.

In particular, the contribution of the social features has been applied to the two main challenges in the selection of a relay among the peer devices, namely the mobility of the devices and the selection of trustworthy peers [2].

One of the biggest problem in traditional peer selection schemes is related to the lack of information regarding the location of other peers to which they send beacons or other probing mechanisms. Usually, this problem is solved through mobility patterns, which predict the position of the devices; however, the mobility of the nodes can be highly variable and then such approaches suffer from accuracy issues: to this, social networking information, which is less variable than node mobility, can improve the reliability in the discovery of a peer for D2D communication, which is willing to relay the content.

In this regard, Zhang et al. [12] and Wang et al. [13] proposed the most recent attempts to take into account social features in D2D communication to foresee a user position. The creation of the link is based on the interaction histories between two users, while at the same time considering information regarding the channel status and the user contact model. A further step in solving the mobility issue through social contribution for D2D communication was given by Zhang et al. [14]. The authors pointed out how connectivity among users is highly intermittent due to the user mobility, leading to the degradation of the QoS (Quality of Service), while social relations are usually stable over time. Social networks are therefore split between offline ones, which only reflect social relations, and online ones, which are a subgroup of offline social networks and consider external influence from media or friends. The authors’ goal was to combine both social networks in order to improve traffic offload when selecting a peer in D2D communications.

Peer selection is usually performed through one of the following two approaches: ad-hoc, where the discovery is made in a distributed way by the devices themselves through the transmission of periodic beacons, and network-controlled, in which a central unit assists the device in the discovery of D2D candidates.

The ad-hoc approach is the most sensible to the mobility issue and it has seen the highest number of contributions from researchers. Zhang et al. [15] suggested the first social approach by exploiting social network characteristics to assist the ad-hoc peer selection. Their goal was to increase the beaconing rate only for a subset of nodes, thus leading to an increase in the detection of their peers. To do this, they first divided the D2D users into communities based on their social features and then selected the nodes with the highest centrality as the subset of nodes. Similarly, the work in [16] proposes a distributed solution for peer selection considering social relationships among users and a dynamic scenario where all users are mobile with the aim to maximize the data rate transmitted to the BS. This idea is based on the work proposed in [17], which shows how two people who share a common friend and have close connection times with him/her have a higher probability to have a similar movement trajectory and therefore are likely to meet and can act as a relay for each other.

All the above-mentioned works are based on the idea that users can only belong to one community at a time. However, due to the multitude of social attributes and interests of people, it is reasonable to assume that a user can belong to more than one community. This is the starting point of the work in [18], which detects overlapping communities and designs a neighbor discovery method for the ad-hoc network approach: the solution identifies the beacon detection rates by dynamically estimating the roles of a user, who belongs to various overlapping communities, to enhance D2D communications both intra and inter-community.

Regarding the network assisted approaches, the authors of [19] proposed a centralized solution based on a coalition formation game to solve the relay discovery problem. Their goal was to push forward the trust and reciprocity derived from social aspects in the D2D communication domain. Similarly, in [20], to improve the stability of D2D communications in a mobile environment, the relay discovery is based on the type of relationship shared between a potential relay and the end user and the relay’s centrality.

The second problem that drives the research for social D2D communication is tied to the relay reliability. Due to the spreading of several wireless devices on the market, especially considering configurable smart ones, choosing wireless terminals as temporary relays may bring data integrity and privacy leakage concerns, thus the selection procedure of trustworthy peers is particularly important for D2D communications.

Several papers study how to exploit social relationships among peers to establish a higher degree of trustworthiness during D2D communications. In particular, in [21], the authors used the social trust levels among nodes to derive a stop-wait approach for the selection of the relay, while, in [22], the goal is to minimize the end-to-end content delivery delay, thus the relay is chosen using both social trust and the propagation link. Social trust is analyzed thoroughly in [23], where the authors studied the objects’ behavior in a 5G scenario to build a reliable system: when a device has to upload content, it selects the relay based on the trustworthiness of its peers. The trust metric is constructed based on the sociality among the devices. Another example is presented in [24], where the authors used a trustworthy social-communication graph to improve the capacity gain and data rate of the D2D communication. Recently, Militano et al. [25] introduced the concept of sociality to identify the presence of malicious node in a network by making use of the concepts of reliability and reputation to model the level of trust among the devices involved in a D2D transmission. Similarly, the authors in [26] proposed a coalition formation game to enhance content uploading services through an opportunistic hop-by-hop forwarding scheme based on the trust of each hop.

However, even if in its infancy, the research towards social networking concepts applied to D2D communications is progressing really quickly, so that some real applications can already be found. In this context, one of the first applications proposed is SoCast [27], which is a video multiCast system that uses social-aware D2D communications. Among the main features of SoCast, the system considers an incentive for the user cooperating in the communication based on the social ties between peers, which is a similar feature to [7] where the relay discovery is based on the concept of reciprocal cycles.

Finally, Nitti et al. [28] proposed an innovative approach for D2D communications applied to a real indoor environment, based on a Social IoT (SIoT) architecture [4] able to involve all participating objects in a twofold procedure, gathering both Quality of Service (QoS) and spectrum sensing data and weighting the received information using a social trustworthiness algorithm.

**Figure 2:**
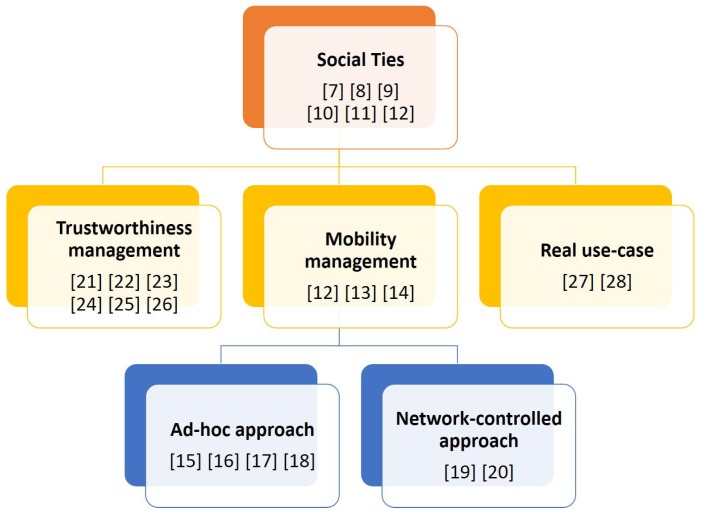
Classification tree.

Figure 2 shows our classification tree for the peer selection approaches based on the two main issues addressed in this context, namely mobility and trust, with the root of the tree aggregating general works applying social concept to the topic.

### 3.2. Communication Mode Selection

During the initial procedure for the socially aware device-to-device (D2D) networks, the decision-making of the transmission mode is a fundamental issue. The choice to select D2D mode or cellular mode for communication is a daunting work in D2D underlaying cellular networks. Communication between two participants, in cellular mode, can only be done via the BS even though both parties of the associated call are in the immediate vicinity. In D2D mode, data packets are transmitted via a direct connection between the user equipments (UEs). Because D2D communication normally uses the same air interface as cellular communication, a UE can only operate simultaneously in D2D or cellular mode [29]. The decision of D2D or cellular communication mode determines, in D2D-enabled cellular networks, the mode selection issue for UEs. A UE needs to establish neighboring UEs as D2D candidates, before mode selection, and perceive their possible service [8]. Given the randomness and variability of content location, the limited transmission and storage capacity of devices, and the coexistence of selfish and altruistic user behaviors, it is important to understand how to promote effective collaboration and how to adapt applicants to content providers to achieve all the benefits of D2D content sharing [30].

Principally, when the D2D, base-station-to-device (B2D) and novel multi-D2D sharing modes exist together, the concern of content sharing mode selection operates the prevalent aspect in this specific matching [31]. Wu et al. [32] presented the concept of socially aware rate, which blends the link rate with the social selfishness from the social knowledge to achieve the effective cooperation together with the physical link quality. Additionally, the sharing mode selection issue based on social awareness is designed as a maximum weighted mixed matching problem, which can be algorithmically diminished to a transposable interest issue subject to a matroid constraint. In [32], the authors developed a framework based on a best-effort distributed algorithm that exhibits possible choice of numerous computational ramifications and estimation rates to satisfy the varied practical demands.

After the mode selection process, the network operator has to allocate the radio resources like frequency bands, time slots, and to transmit the D2D and cellular links, respectively. Those issues of nearby UE detection, wireless resource allocation and communication mode selection are coupled with each other, and are always connected with level loading over a network, the user-level behaviors and channel conditions, thus delivering new challenges for cellular networks [33]. Recent studies have identified that, by using the cellular resources in a D2D communication underlaying cellular system, UEs can determine to transfer data over their serving BSs, or to communicate with each other via direct links [15,34]. By achieving the awareness of the instantaneous network load, potential D2D pairs and channel conditions, the system can choose the best mode, assign the resources and handle interference efficiently, to recognize the proximity, reuse and hop gains delivered by D2D communication [35,36]. Mode selection relies on the awareness and recognition of the intercell interference, channel condition, and network load. Thus, managing the community structure information can facilitate the discovery of the aforementioned three physical parameters to make the mode selection decision accurately and quickly for the user [37].

Li et al. [38] presented a solution to increase the data transmission volume from all the BSs to all the UEs. To better monitor the channel condition, the system generally has to examine security and privacy concerns. The social tie information can be analyzed as a way to conclude the trust between two peers, because the transmission power of a tie can be associated to the reliability of the channel between two nodes. Hence, in the D2D communication system, managing the data and knowledge of social ties in resource allocation can be used not only to achieve higher throughput distinguished to an architecture that is non-socially-aware but also enhanced security and privacy [39].

To bypass possible network congestion, the resource allocation and mode selection processes could be aware also of the community bridges. Particularly, the resource allocation modules need to allocate more resources to the users of the bridge, as long as the mode selection module has to deliver high preference for cellular communication to bridge peers. The bridge user detection algorithms, bridge-aware resource allocation and mode selection schemes have the potential to enhance and increase the global coverage and throughput of the D2D communication underlaying cellular network [38].

An interesting idea for autonomous mode selection was presented by Kumbhar et al. [24] who combined relay discovery schemes and communication graph to establish social relationships (social graph). The solution, called Reliable Relay, also includes a self-governing archetype, Social D2D (S-D2D), by adding trust factors in mode selection schemes. Performance observation and interpretation suggest that the proposed Reliable Relay scheme increases capacity gain and data rate with imperceptible delay.

On another research topic, Hoeyhtyae et al. [40] presented a study of power-efficient transmission mode selection for social-aware D2D communications in future networks aiming at integrating WiFi and LTE air interfaces seamlessly. Various smartphones have diverse power profiles and thus the optimization method should take this information into account. Power consumption models for LTE and WiFi interfaces commonly have a static part just for being active and a linear rate-dependent part. In the most recent models, the static part is clearly the dominating factor, because the increase in the data rate increases the power consumption lightly, as demonstrated in [40]. Hence, it is suggested that a heterogeneous mobile social network tries to minimize the number of active interfaces to save power. It would be beneficial if the smartphones could be developed so that the static part in power consumption would be close to zero and otherwise the communication based consumption would be a linear function of the bit-rate for a given distance. This would enable better optimization of transmission power for distinctive scenarios and schemes.

When social connections among users are exploited in a way that fairness is ensured, the awareness of social characteristics could be advantageous also for the Quality of Service (QoS) of D2D cooperative communication. The communication mode selection and peer selection problem arise, at the D2D cooperative network creation stage, as users suitable to engage in D2D cooperation must be identified. However, in practice, the users’ hesitation to cooperate by allowing access to their equipment to others or granting the distribution of their own data among other devices, can still create difficulty in D2D communication [41].

Huynh et al. [42] introduced a socially aware energy efficiency optimization solution for D2D communications in 5G networks, in which the energy efficiency optimization issue is answered for optimal channel mode selection and optimal transmission powers allocated to each mobile user (MU) in order to boost the energy efficiency, by making use of adaptive genetic algorithm. Thus, the increasing of social networks (e.g., Twitter, Facebook, etc.) can give favorable opportunities to develop and enhance the performance of D2D communications in 5G networks.

**Figure 3:**
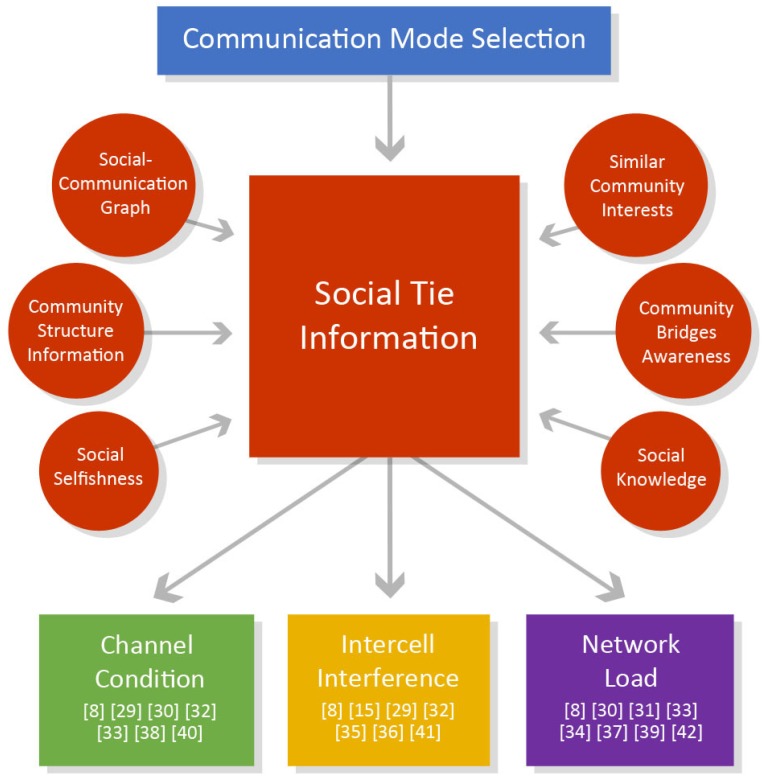
Enhancing the mode selection decision using social tie information.

Figure 3 presents an overview on the current research directions for the communication mode selection, with an overview of the references for each specific direction.

In conclusion, the communication mode selection can include various social ties to be used for managing channel condition, intercell interference and network load, based on the following social concepts: similar community interests, community bridges awareness, social knowledge, social selfishness, social-communication graph and community structure information.

### 3.3. Spectrum Resource Allocation and Management

In this section, all significant works concerning resource allocation and optimization issues for D2D communications are shortly described. Some of these can be grouped considering similar approaches, methods or algorithms. Six main key requisites to design social-aware resource allocation and optimization for D2D communication were identified [43]:

- Social-aware metrics: Considering the high variability of social-aware D2D communications both in time and in the type of applications and relationships between users, allocation mechanisms need to assure dynamic generalization and adaptation.
- Scheduling: Dynamic scheduling algorithms are crucial to improve resource allocation evaluating the resources necessary to set the users’ application demands and assigning them.
- Resource Heterogeneity: It is in terms of device spectrum diversities (e.g., time variance of the spectrum and features of channels) and capabilities (e.g., throughput, energy, and interfaces helpful for transmission).
- Resource Optimization: These kinds of mechanisms can assure the usage of the available resources in a better way, meeting the required service level accordingly.
- Estimation Accuracy: Social interaction between devices needs to carefully manage the monitoring of social-aware D2D links.
- Privacy: It is crucial to be assured by enabling device protection and social information protection (e.g., encrypting data and deploying a rigid policies concerning access personal information).

We start our analysis from the social-aware metrics, for which Li et al. [38] proposed in 2014 a social-aware enhanced D2D communication system to find solutions to the main D2D communications issues, such as mode selection, interference control, and allocation of resources. Resources (i.e., transmission power, frequencies, and channel conditions) need to be efficiently managed to preserve D2D and cellular communications from mutual interference because interference level is essential to assure the closeness and reuse. Another example is the work in [44], where the authors applied the social relationships among the objects to enable an efficient and trustworthy spectrum utilization in a cognitive radio scenario. In [45], an advance of the Bayesian theory is exploited to achieve the social ties between devices that are offline in mobile networks based on their social activities. The authors described a method to learn relationships using a coalitional graph game: the number of served users grows according with their popularity. Zhao et al. [46] presented a game theory model to improve the serviceability perceived by D2D users of a social group. The main objective was to find the Nash Equilibrium (NE) of the maximization game performing a distributed allocation of resources. Both D2D and mobile users are characterized by an utility function based on social features. The way of structuring the game makes the equilibrium complicated to be always reached. In [47], the authors formulated the distributed power and subcarrier allocation problem in a D2D content dissemination system as an evolutionary game. A new algorithm for global search was carried out to obtain the evolutionary equilibrium considering the predicted contact duration to limit computation complexity and data exchange. The eNB studies the framework of the social network examining the related platform (e.g., Facebook and Twitter) to calculate social trajectory similarities and to send them to D2D users. Successively, D2D users choose transmission adjustment and calculate their services in a random way and send their reports to eNB. Thanks to these information, the eNB can select the average utility in order to broadcast it to all D2D links. In this way, D2D links are able to evaluate and decide for utility changing until the game equilibrium achievement. In [48], an optimal social-community aware resource allocation (OSRA) algorithm for D2D communication is developed for limiting the duration of D2D communications taking advantage of users’ social characteristics. The solution shows a centralized nature that allows a reduction of data transmissions but overloads the BS. In [8], the authors faced the increasing storage space problem, proposing to let the distributed file caches be exploited by cellular networks, replacing cellular links with local D2D links in order to decrease mobile traffic and to improve spectrum utilization. While a D2D device is caching information desired by several adjacent UEs, a procedure to create a cluster for multicast communications can start [48]. The bandwidth is dedicated to the cluster head. The proposed Social Aware-D2D (SA-D2D) scheme allows a cluster head to perform in an asynchronous way to exchange information using the same spectrum, guaranteeing goodness of transferred videos. To do this, two approaches were presented: a half duplex (HD) and a full-duplex (FD) approaches. A half-duplex approach in which two orthogonal time stages are needed [49]. In the first one, considering a cluster, all UEs can obtain information transmitted by the BS over the mobile downlink. Successively, the cluster head is able to multicast data to the other UEs over direct D2D links inside the cluster. With the full-duplex approach, a cluster head communicates on the same frequency band in a simultaneous way (i.e., transmission and reception), but it must use two different antennas (i.e., one for transmission and one for reception). Both self-interference and co-channel interferences can be mitigated using existing methods [50,51].

In the literature, several works propose solutions to cope with the scheduling issues. In [52], data exchange is studied in terms of inter-community and intra-community data forwarding. Neighbor users (i.e., physically close) can have very different social relationships and this information can be used to influence short connections, also leading to an underutilization of resources. For this reason, a cooperative approach can help users in considering both social and physical attributes, to select the optimal way to cooperate for reducing delay. Both a coalition game and the concerns of users are used to divide them into fog communities, leading to a new, highly scalable network architecture. In [27], the authors presented a distributed algorithm based on a coalition game to obtain clients grouping for video content multicast distribution in a cooperative way. A resource allocation module was carried out to improve BS management of D2D links in order to maximize utilization of resources. The service delivery is based on the restoration of video frames. In this context, the BS take decisions on resource allocation according to the received requested from each group, not needing to previously know the video features and the social relationships between clients. In the implementation of multicast dissemination, the interference can be managed with Base Station and Access Point control, as proposed in [53]. Efficient scheduling and radio resource allocation can be improved using network information (e.g., channel condition, potential D2D pairs, etc.) increasing both signal overhead and, at the same time, efficiency of local data dissemination.

Resource heterogeneity is another challenging line for social-aware communications. In [54], a two-step coalition game was developed to efficiently allocate resources to D2D communications considering uplink channels of a cellular network. The evaluation of the proposed framework was performed in real human traces and random networks showing its effectiveness achieving a substantial improvement in performance compared to systems that do not provide resource management based on community aware methods. A time-varying social-aware resource allocation for D2D communication has been proposed in [55], based on the Auto-Regressive Integrated Moving Average (ARIMA) model with the call records. Social community is analyzed to obtain an utility function for a potential game. The authors demonstrated that the new algorithm reaches NE, improving the total utility of the system and increasing system capacity. In [22], several new relaying strategies are presented and described. For example, the relay discovery strategy allows multiple relay nodes to choose the best pair of source and destination channels, introducing a feedback procedure used by the destination to give information about the transmission. Full duplex strategy assures higher spectral efficiency but, as a drawback, it shows loop interference and self-interference in the presence of relays with multiple antennas. Instead, half-duplex relays reveals multiplexing losses. Sakr et al. [56] presented an energy-harvesting-based D2D communications system model where the D2D transmitter harvests energy from environment interference and communicates with its D2D corresponding receivers over channels allocated to cellular users. In the device relaying and multicast scenarios, energy harvesting can be taken into account as an efficient incentive mechanisms, due to the possibility for the devices to use the harvested energy to broadcast or multicast local contents instead of their own power [53].

Many algorithms tackle the resource optimization problem. Yu et al. [57] analyzed methods to share resources maximize the throughput in D2D communications performed in cellular networks. In [58], a power optimization framework managing interference from cellular users by proposing a δD-interference limited area control scheme is proposed. In [59], the authors considered peer and service discovery as the first step to start D2D communications. Device relaying and multicast dissemination scenarios are proposed, where devices requesting local traffic can be performed in dedicated portion of band or reusing licensed spectrum. Clearly, in the second case, interference problems need to be carefully managed, even if the spectrum is used more efficiently. In [60], D2D transmissions in downlink OFDMA networks are considered. A two-hop socially-aware method for resource allocation is presented: a device is connected with the base station (i.e., first hop) and can also perform a new communication with another device (i.e., second hop). This solution shows disadvantages when the number of iterations between the first hop and the other devices increases. In [61], the authors modeled D2D communications in terms of sociality finding a new characteristic of link strength, that is the contact time. The contact time was set as a constraint to limit the transmitting time of a D2D communication. Performance of network is influenced by both physical features (e.g., number of user) and social features (i.e., contact time). The sociality-aware scheme improves network throughput thanks to the contact time. In [62], routing algorithms over peer-to-peer (P2P) share enabled multi-hop D2D networks are proposed. D2D users try to download common data from several distributed D2D servers. The Routing scheme with Location-Aware P2P-Share (R-LPS) mechanism is presented: the main objective is to calculate a route passing by or directly through other users. Simulation results demonstrated that, for the generalized case, all the schemes allow improvement in terms of throughput. In [63], a relay node can be helped to increase the spectral efficiency. This method allows choosing the best buffered relay between all destination links, for transmitting data if channel is good or, alternatively, buffer it. In a D2D relay network, one of the mobile devices in a D2D communication device pair is called a relay device, able to perform as a forwarder for the BS in case the perceived quality of the transmission from the BS to the end user is under a satisfying level [20]. In this way, the throughput perceived by a higher number of users can increase, as well as the coverage area of a base station not introducing co-channel interference to devices in other cells [64].

Finally, some works focus on estimation accuracy. In [38], qualitative and quantitative evaluations are performed to assess performance growing by allowing social-aware D2D communication. In [42], the energy efficiency optimization (EEO) problem in D2D communications is formulated, analyzing both the social conjunctions and physical interference among mobile users. The starting idea is to allow D2D pairs in sharing spectrum resources of a cellular subscriber to increase spectrum efficiency. An adaptive genetic algorithm is applied to solve this EEO problem to maximize the energy efficiency, while at the same time assuring high system throughput and spectrum efficiency considering QoS constraints. In [65], the authors analyzed a relay-aided D2D network focusing on wireless channels uncertainty in order to provide a stable, optimal, and unique solution. The proposed method is close to the centralized optimal solution, showing a low level of computational complexity.

Actually, it seems that privacy is an open issue and no relevant works have been found in the literature. Similarly, social information protection is a hot topic also considering the growing interest of users in defending their personal data. In fact, in the last two years, legal order started a process to improve laws and fundamental rights all over the world.

**Figure 4:**
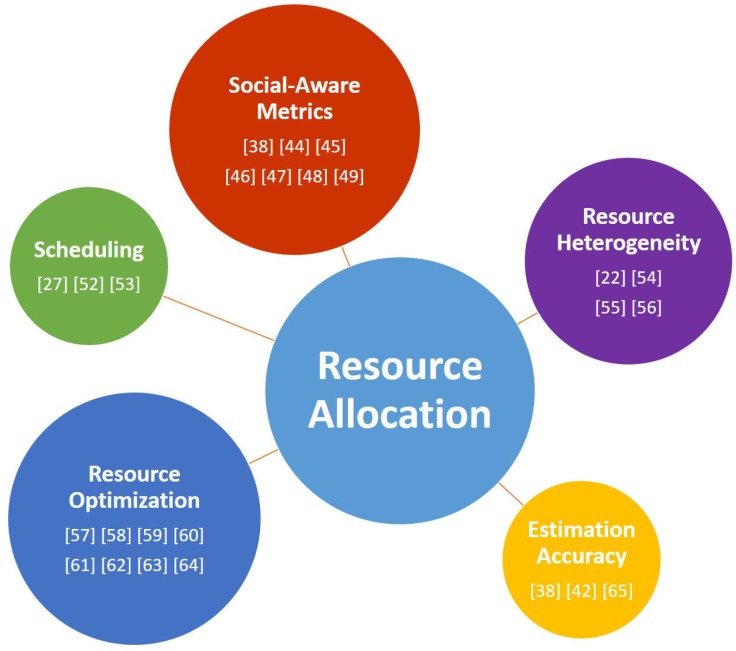
Resource allocation works grouped considering key requirements to design social-aware resource allocation and optimization for D2D communication.

In Figure 4, all papers presented in this section are grouped and summarized. The dimension of circles is proportional to the number of found references.

## 4. Lesson Learned

In the past years, social network concepts have been applied to D2D communications. The convergence between social networks and D2D communication is still in its infancy, however the already available scientific contributions clearly demonstrate that the social contribution is fundamental for the improvement of D2D communications. This section overviews the existing gaps in the research and points out the current challenges that still must be addressed.

Several works use the concept of sociality, however there is no common agreement regarding the origin of such sociality, whether it should be inherited from the social networks of people or created among the devices themselves. Future research should therefore address this difference, since the resulting social networks, among people or things, have different characteristics and can then be more or less useful based on the issue at hand.

Among the issues in which the social concepts have been applied, the relay discovery and peer selection topic has been deeply investigated. In particular, the usefulness of social networks has been proven in helping the devices involved in a D2D communication to select reliable and trustworthy peers as their relays, and identify and avoid any malicious device. Another problem tackled by the research community is the prediction of the user’s location: even if the number of papers studying this issue is large, we believe that further studies are needed to assess the contribution of sociality, since until now the benefits of social networks to foresee the user’s mobility have only been tested through simulations in very specific scenarios, and the literature is still missing experiments on large, real datasets concerning aspects of people and objects behavior. Finally, researchers should concentrate their efforts to apply these concepts to real use cases, since only a couple of works have tried to explore this territory.

The communication mode selection topic has seen several social concepts applied to it. To achieve the effective cooperation together with assuring the physical link quality, the concept of socially aware rate can be used to blend the link rate with the social selfishness from the social knowledge. The new challenges for cellular networks can be managed and handled using community structure information and user-level behavior, improving the management of the channel condition, the inter-cell interference, and reducing the network load. For this specific topic, we believe that there is the need for more research on real applications and also for the development of more realistic scenarios to identify and establish the distinctness between the test use cases and real use cases using the social knowledge and social selfishness.

Combining relay discovery schemes and communication graph to establish social relationships can be used when social connections among users are exploited in a way that fairness is ensured, enhancing also the Quality of Service with the use of social characteristics.

The perspective of socially aware communication mode selection will probably be emergent and unpredictable, and there is the need to achieve a better understanding of social relationships and of the automatic selection of connections from a group of social characteristics focused on the community structure information and similar community interests.

The resource allocation topic has also been analyzed since the beginning of the social-aware approach. Five metrics are presented to summarize the main challenges faced in the last years by research community to improve resources utilization in order to optimize their management considering the growing demand in terms of number of users and services. The proposed strategies do not match a common thread, even when taking into account the same metric, leading to some interesting result, limited though to a specific network or to a particular algorithm. Many specific solutions can be found in the literature, nevertheless a generally recognized direction for the management and reuse of resources is still missing, so it can be largely considered an open topic. Finally, the literature is still lacking solid works to ensure privacy in social-aware resource allocation, which remains an open issue, especially in light of the introduction of international guidelines such as the General Data Protection Regulation (GDPR) introduced in 2018 [66].

## 5. Conclusions

In this paper, a comprehensive study of the convergence between social networks and D2D communication found in the literature is presented. The concept was proposed around 5–6 years ago: the number of papers, even if limited, is still relevant when compared to this time frame, showing that this topic clearly represents an emerging trend. We analyzed this convergence under three different aspects: relay discovery and peer selection, communication mode selection, and spectrum resource allocation and management.

The main aims of this survey are to investigate the benefits of the integration of social-awareness with D2D communication, and to give to the reader a clear picture of the current scientific efforts on the D2D “communication domain”. From the analyzed papers, it clearly emerges how social networks can improve D2D communications under different aspects. However, the efficiency of the proposed solutions has to be proven with the aid of real use cases.
